# 3M syndrome with novel *CUL7* variants in a Chinese patient: a case report

**DOI:** 10.3389/fped.2025.1652826

**Published:** 2025-10-29

**Authors:** Hui Liu, Gaojie Liu, Weicai Suo, Shuaishuai Han, Yizhong Wang, Hongfang Ding

**Affiliations:** ^1^ Department of Pediatrics, Shengli Oil Field Central Hospital, Dongying, Shandong, China; ^2^ Department of Gastroenterology, Hepatology, and Nutrition, Shanghai Children's Hospital, School of Medicine, Shanghai Jiao Tong University, Shanghai, China; ^3^ Gut Microbiota and Metabolic Research Center, Institute of Pediatric Infection, Immunity and Critical Care Medicine, School of Medicine, Shanghai Jiao Tong University, Shanghai, China

**Keywords:** 3M syndrome, *CUL7*, short stature, dysmorphic facial features, skeletal anomalies

## Abstract

**Background:**

3M syndrome is a rare autosomal recessive disorder caused by biallelic pathogenic variants in the cullin 7 (*CUL7*), obscurin-like 1 (*OBSL1*), and coiled-coil domain-containing protein 8 (*CCDC8*) genes and is characterized by pre- and postnatal growth retardation, short stature, dysmorphic facial features, and skeletal anomalies, with normal intelligence.

**Case presentation:**

In this study, we report a 6-year-old female patient from China diagnosed with 3M syndrome. The patient presented with typical clinical features of growth retardation and short stature, with normal intelligence. The patient’s dysmorphic facial features included relative macrocephaly, a protruding forehead, a triangular face, a pointed chin, a flat nasal bridge, full lips, a long philtrum, and a broad lower jaw. The skeletal survey was normal except for clinodactyly of the fifth fingers of both hands. Growth hormone (GH) deficiency was excluded by normal serum hormone levels and the GH stimulation test results. Whole-exome sequencing identified two heterozygous variants in *CUL7*, NM_014780.5: c.1639_1640del (p.Leu547Alafs*6), and NM_014780.5: c.4505T>C (p.Ile1502Thr). Parental Sanger sequencing confirmed these as compound heterozygous variants, with one variant inherited from each parent. Neither variant has been previously reported. The patient has been treated with recombinant human IGF-1 for 2 years since she was 4 years old and has achieved a growth velocity of approximately 6–7 cm per year.

**Conclusions:**

Herein, we describe a Chinese patient with 3M syndrome caused by novel biallelic pathogenic variants in *CUL7* from a non-consanguineous family, expanding the genetic spectrum of *CUL7* in the Chinese population.

## 1 Introduction

3M syndrome is a rare autosomal recessive primordial growth disorder characterized by severe pre- and postnatal growth retardation, characteristic facial features, skeletal anomalies, and normal intelligence. It was first described by Miller et al. in 1975 (1). Patients with 3M syndrome are typically born with low weight and short length, leading to a short stature in adulthood (2). The dysmorphic facial features include relative macrocephaly, a protruding forehead, a triangular face, a pointed chin, a flat nose, full lips, and a long philtrum. Skeletal dysplasia, such as a short and broad neck, square shoulders, short thorax, pectus carinatum or excavatum, winged scapulae, scoliosis, clinodactyly, prominent heels, and joint hypermobility, may present in affected individuals (3). The exact prevalence of 3M syndrome is unknown, with approximately 250 cases reported in the literature (4). According to the molecular etiology, it is divided into three forms, namely, 3M syndrome-1 (OMIM #273750), 3M syndrome-2 (OMIM #612921), and 3M syndrome-3 (OMIM #614205), which are caused by biallelic loss-of-function variants in the cullin 7 (*CUL7*), obscurin-like 1 (*OBSL1*), and coiled-coil domain-containing protein 8 (*CCDC8*) genes, respectively (3). *CUL7* is the predominant pathogenic gene, accounting for approximately 65% of the patients with 3M syndrome, followed by *OBSL1* (around 30%), and less commonly in *CCDC8* (5). However, the pathogenic variants of these three genes do not account for 100% of individuals with 3M syndrome, indicating that other genes may be involved. The underlying mechanism is not clearly understood; pathogenic variants in the *CUL7*, *OBSL1*, and *CCDC8* genes are thought to disrupt the 3M complex formed by the CUL7, OBSL1, and CCDC8 proteins, which play critical roles in maintaining microtubule and genome integrity and in normal development (6).

Currently, only a few individuals with 3M syndrome from China have been reported in the literature. In this study, we report a female pediatric 3M syndrome case from a non-consanguineous Chinese family with the novel *CUL7* compound heterozygous variants NM_014780.5: c.1639_1640del (p.Leu547Alafs*6) and NM_014780.5: c.4505T>C (p.Ile1502Thr), expanding the genetic spectrum of *CUL7*-associated 3M syndrome in the Chinese population.

## 2 Case presentation

A 3-year-old female child was referred to the Department of Pediatrics, Shengli Oil Field Central Hospital for an evaluation of her short stature. The female child was born at full term via normal vaginal delivery with a birth weight of 2.49 kg [−2.24 standard deviation (SD)] and a birth length of 46 cm (−2.18 SD). The postnatal period was uneventful, and she was intellectually normal. She was the second child of healthy non-consanguineous parents. The family history was unremarkable. The mother's height was 157 cm, the father's height was 181 cm, and her 9-year-old elder sister was 140 cm tall. At age 1 year and 6 months, the parents noticed that the patient presented with growth delay, and her height was significantly shorter than that of other children of the same age.

**Figure 1:**
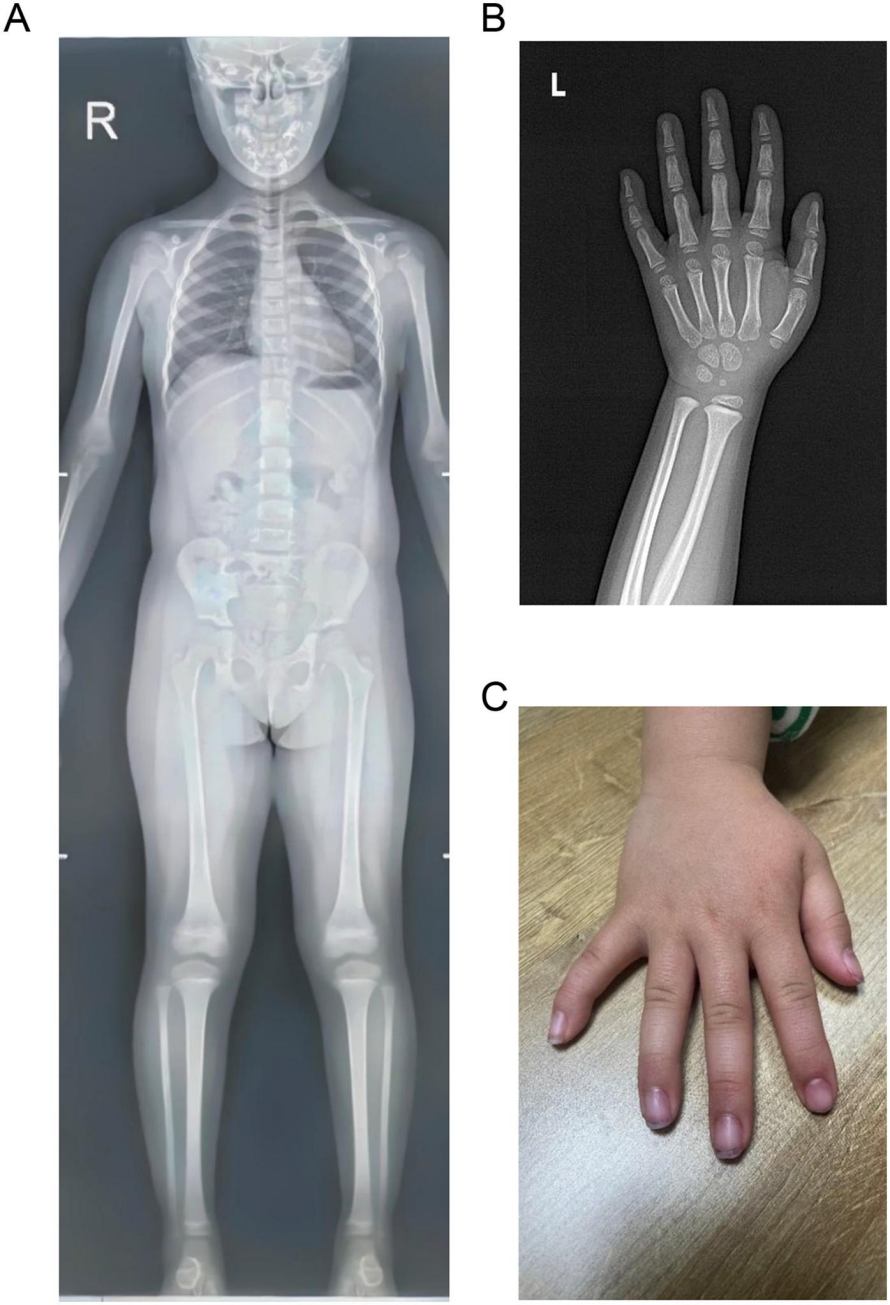
Skeletal survey images of the patient. **(A)** Normal anteroposterior x-ray image. **(B)** X-ray image of the left hand showing clinodactyly of the fifth finger. **(C)** Image of right hand showing clinodactyly of the fifth finger.

On admission, the physical examination showed that the weight and length of the patient were 10 kg (−3.20 SD) and 82.5 cm (−3.63 SD), respectively. Her body temperature was 36.5°C, heart rate was 110 beats per minute, respiratory rate was 25 breaths per minute, and blood pressure was 78/56 mmHg. The cardiopulmonary and abdominal examinations were unremarkable. However, she had relative macrocephaly, a protruding forehead, a triangular face, a pointed chin, a flat nasal bridge, full lips, a long philtrum, and a broad lower jaw. The skeletal survey using x-rays showed a normal skeleton except for clinodactyly of the fifth fingers of both hands (Figure 1).

The initial hormonal tests revealed normal levels of insulin-like growth factor 1 (IGF-1; 104.11 ng/mL, reference range: 49–311 ng/mL), free triiodothyronine (T3; 3.85 pg/mL, reference range: 2.41–5.50 pg/mL), free thyroxine (FT4; 0.98 ng/dL, reference range: 0.96–1.77 ng/dL), and thyroid-stimulating hormone (TSH; 2.18 μIU/mL, reference range: 0.70–5.97 μIU/mL). The patient’s serum fasting blood glucose (FPG; 4.84 mmol/L, reference range: 3.89–6.11 mmol/L), fasting insulin (FINS; 67.00 pmol/L, reference range: 13–161 pmol/L), and glycated hemoglobin (HbA1c; 5.3%, reference range: 4%–6%) levels were normal. The growth hormone stimulation test (GHST) using arginine and levodopa peaked at 90 min (16.01 ng/mL), and GH deficiency was excluded. After discharge, a conservative approach that focused on improving diet and lifestyle was taken to achieve catch-up growth as the patient was small for gestational age (SGA). The patient started to receive recombinant human IGF-1 (rhIGF-1) therapy (0.2 mg/kg/week) because of her growth delay at 4 years old (height: 92 cm, −2.92 SD; weight: 12.5 kg, −2.33 SD). Her growth velocity was approximately 6–7 cm/year during the following 2 years of continuous rhIGF-1 therapy. She was 6 years old at the last examination with a height of 106.5 cm (−2.25 SD) and a weight of 20 kg (−0.15 SD), and her intellectual development was normal.

**Figure 2:**
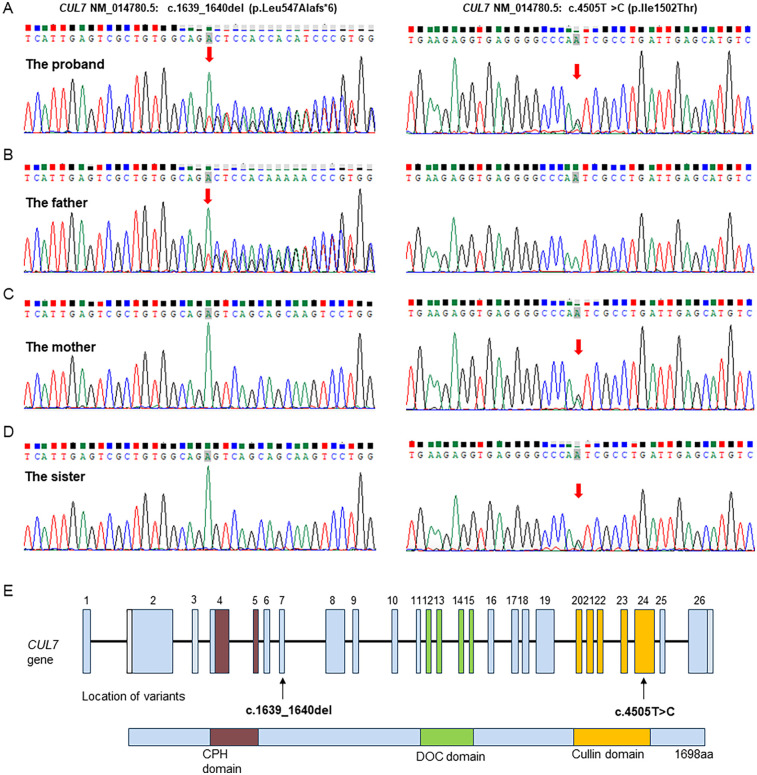
Sanger sequencing validation of the *CUL7* variants identified by whole-genome sequencing in the family. (**A**) The proband carried both the NM_014780.5: c.1639_1640del (p.Leu547Alafs*6) and NM_014780.5: c.4505T>C (p.Ile1502Thr) variants. (**B**) The father carried the NM_014780.5: c.1639_1640del (p.Leu547Alafs*6) variant. (**C**) The mother carried the NM_014780.5: c.4505T>C (p.Ile1502Thr) variant. (**D**) The healthy older sister carried the NM_014780.5: c.4505T>C (p.Ile1502Thr) variant. (**E**) Visual representation of the identified *CUL7* variants.

**Figure 3:**
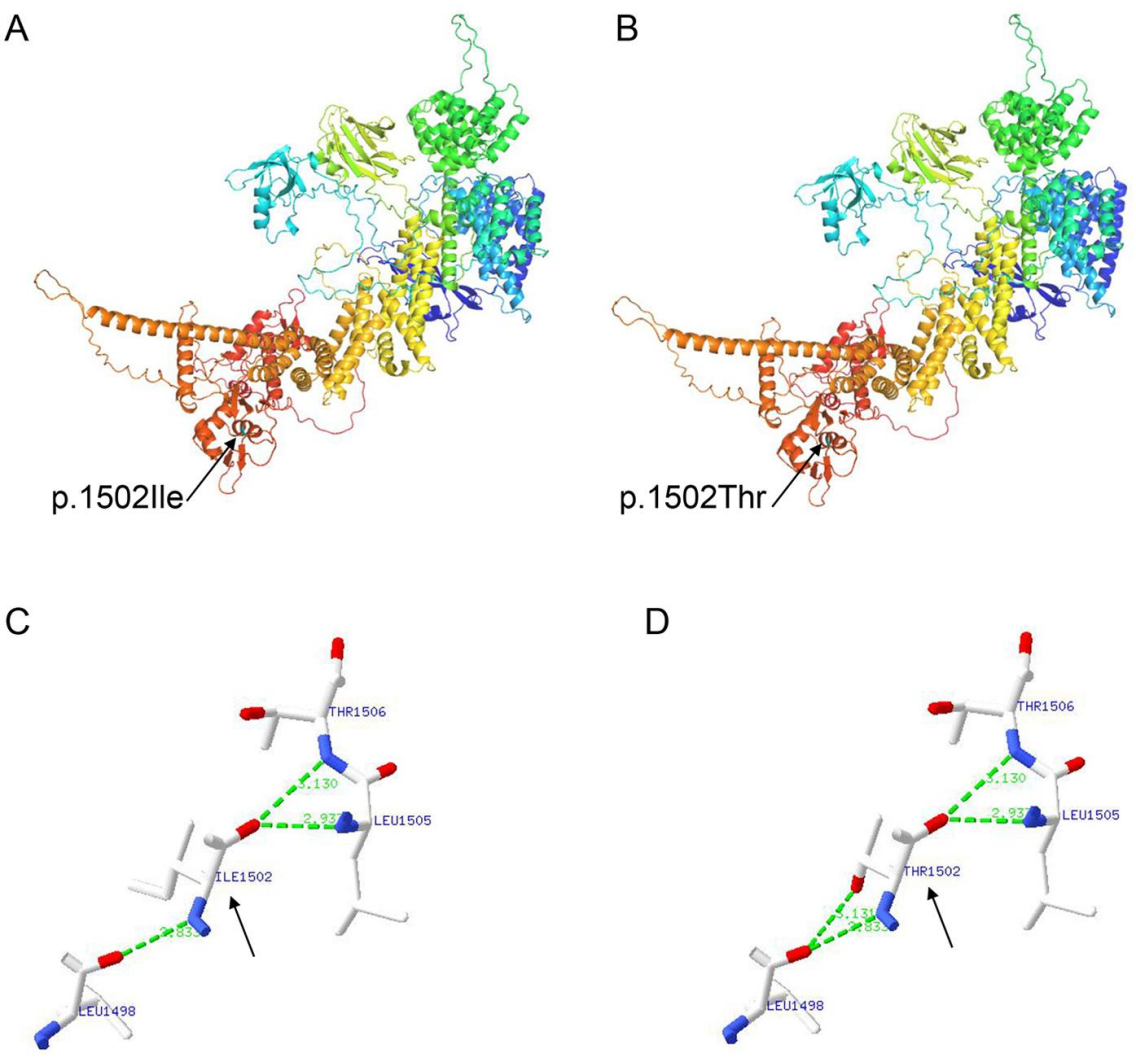
An *in silico* structural analysis of the CUL7 protein using AlphaFold2. (**A**) The structure prediction of the wild-type CUL7 protein. (**B**) The structure prediction of the NM_014780.5: c.4505T>C (p.Ile1502Thr) CUL7 protein. (**C**) A partial enlargement of the wild-type CUL7 protein. (**D**) A partial enlargement of the NM_014780.5: c.4505T>C (p.Ile1502Thr) CUL7 protein.

To evaluate the genetic cause of her short stature, whole-exome sequencing (WES) was performed using genomic DNA extracted from the patient’s peripheral blood. The WES identified two novel variants, namely, NM_014780.5: c.1639_1640del (p.Leu547Alafs*6) and NM_014780.5: c.4505T>C (p.Ile1502Thr) in the *CUL7* gene, which were further confirmed using Sanger sequencing (Figure 2A). Further genotyping of the unaffected parents using Sanger sequencing revealed that the NM_014780.5: c.1639_1640del (p.Leu547Alafs*6) variant was present in the father (Figure 2B) and the NM_014780.5: c.4505T>C (p.Ile1502Thr) variant was present in the mother (Figure 2C), confirming that the proband carried compound heterozygous *CUL7* variants. Her healthy older sister carried the NM_014780.5: c.4505T>C variant (Figure 2D). The location of the identified *CUL7* variants is shown in Figure 2E. The NM_014780.5: c.1639_1640del (p.Leu547Alafs*6) variant results in a premature termination codon, and the aberrant transcript will likely be degraded by non-sense-mediated mRNA decay (NMD). The NM_014780.5: c.4505T>C variant results in the amino acid substitution of threonine for isoleucine at codon 1502 (p.Ile1502Thr). An *in silico* prediction of the NM_014780.5: c.4505T>C variant using the rare exome variant ensemble learner (REVEL) method showed a score of 0.542 (<0.65) (7). Neither variant has been reported in the 1000 Genomes Project database or the Genome Aggregation Database (gnomAD) nor in previous literature. The NM_014780.5: c.1639_1640del (p.Leu547Alafs*6) variant is defined as likely pathogenic (PVS1 + PM2_Supporting), and the NM_014780.5: c.4505T>C variant (PM3+PM2_Supporting) is defined as a variant of uncertain clinical significance according to American College of Medical Genetics and Genomics (ACMG) guidelines (8). An *in silico* analysis of the three-dimensional protein structure of the CUL7 protein using AlphaFold2 indicated that variant c.4505T>C causes the amino acid at position 1,502 to be mutated from isoleucine to threonine, leading to an additional hydrogen bond between isoleucine at position 1,498 located in the *α*-helix region of the CUL7 protein (Figure 3). Taken together, the diagnosis of 3M syndrome-1 caused by novel compound heterozygous variants in the *CUL7* gene was made in this patient. Currently, she is being managed with continuous rhIGF-1 therapy (0.21 mg/kg/week) with close follow-up, and has grown 2 cm in the last 3 months.

## 3 Discussion

Although patients with 3M syndrome present a unique set of clinical features, including growth retardation, dysmorphic facial features, and skeletal anomalies, the range and severity of the manifestations are very variable (4). In 2025, Elsayed et al. conducted a comprehensive update review of the clinical and molecular characteristics of 3M syndrome (3). The most striking manifestation of 3M syndrome is severe pre- and postnatal growth restriction, which often results in short adult height 4–6 SDs below the mean without catch-up growth (4, 5). The craniofacial features of individuals with 3M syndrome are characterized by relative macrocephaly, a triangular face with a protruding forehead, a flat midface, a flat nose, a long philtrum, and full lips (4). A total of 14 Chinese children diagnosed with *CUL7* variant-caused 3M syndrome (excluding 4 prenatal cases) have been reported in the literature (9–15). Overall, no specific genotype-phenotype associations have been found in Chinese patients with 3M syndrome caused by *CUL7* variants (Supplementary Table S1). In this report, the patient presented with prenatal growth failure with a low birth weight below the mean and showed the typical craniofacial features of relative macrocephaly, a protruding forehead, a triangular face, a pointed chin, a flat nasal bridge, full lips, a long philtrum, and a broad lower jaw. However, inconsistent with other patients with 3M syndrome (16), the physical examination and radiographic imaging revealed no musculoskeletal abnormalities in the patient except for clinodactyly of the fifth fingers of both hands.

3M syndrome is phenotypically homogeneous but genetically heterogeneous. The molecular etiology can be attributed to biallelic pathogenic variants in the *CUL7*, *OBSL1*, and *CCDC8* genes, and the syndrome is often found in consanguineous families (4). *CUL7* is the most prevalent pathogenic gene, and pathogenic variants in *CUL7* have been identified in more than 65% of patients with genetically confirmed 3M syndrome (5). The exact mechanisms of how the pathogenic variants in these three genes lead to the clinical features of 3M syndrome remain unclear. The CUL7, OBSL1, and CCDC8 proteins form a CUL7-OBSL1-CCDC8 complex in the ubiquitin-proteasome system, which plays a critical role in multiple cellular processes, including cell cycle control, apoptosis, signal transduction, and the regulation of microtubule dynamics and genome integrity (6, 17). It is postulated that a loss of ubiquitination is the main underlying pathological mechanism in 3M syndrome (18, 19). Functional experiments have shown that the CUL7 E3 ubiquitin ligase complex targets a key molecule in insulin/IGF-1 signaling, insulin receptor substrate 1 (IRS-1), to control cellular growth (20). The disruption of the CUL7 E3 ubiquitin ligase complex induces cellular senescence, which contributes to the growth restriction in 3M syndrome (21). In this study, two novel variants, namely, NM_014780.5: c.1639_1640del (p.Leu547Alafs*6) and NM_014780.5: c.4505T>C (p.Ile1502Thr), in the *CUL7* gene were identified in the family. Neither of the variants has been reported previously, with NM_014780.5: c.1639_1640del (p.Leu547Alafs*6) defined as likely pathogenic and NM_014780.5: c.4505T>C (p.Ile1502Thr) as a variant of uncertain clinical significance. An *in silico* analysis of the three-dimensional structure of the CUL7 protein revealed that the NM_014780.5: c.4505T>C (p.Ile1502Thr) variant causes an additional hydrogen bond between isoleucine at position 1,498 located in the *α*-helix region of the CUL7 protein. An increase in hydrogen bonds may cause the protein to have an abnormal helical structure, leading to spatial changes in the protein’s structure. Nevertheless, further functional studies are required to further clarify the pathological mechanisms in 3M syndrome.

Currently, there are no available clinical practice guidelines for the management of 3M syndrome. Multidisciplinary supportive treatments are recommended to improve the quality of life of the patients, optimizing their growth potential and reducing skeletal complications (4). First, the presence of a GH deficiency should be determined at the time of diagnosis by an endocrinologist. Several studies have shown that GH levels are usually normal in children with 3M syndrome, and the majority of affected individuals had sufficient peak GH responses (2, 5, 22). Indeed, our patient had normal GH levels and a sufficient peak GH response. The administration of GH therapy is still controversial in children with 3M syndrome due to its variable efficacy. Clayton et al. reported a significant increase in both height velocity and height following 1 year of recombinant human growth hormone (rhGH) treatment in 16 affected children (2). Other studies showed that GH therapy may be effective in the stimulation of catch-up growth in patients with 3M syndrome (16, 23, 24). Karacan Kucukali et al. reported a good early response, which decreased in the following years during long-term GH treatment (25). In contrast, other studies have reported that some affected patients fail to respond to the GH therapy (22, 26). Our patient received rhIGF-1 therapy at 4 years old and achieved a growth velocity of 6–7 cm/year during the following 2 years. Furthermore, other supportive treatments for musculoskeletal manifestations may be administered, such as orthopedic treatments for hip dysplasia/dislocation and kyphoscoliosis.

## 4 Conclusion

In summary, this case report described a Chinese patient with 3M syndrome caused by biallelic pathogenic variants in *CUL7* from a non-consanguineous family. Pediatricians should be aware of this rare and underdiagnosed disease, and a genetic test is necessary to make a diagnosis in children with growth retardation of unknown etiology.

## Data Availability

The original contributions presented in the study are included in the article/Supplementary Material and further inquiries can be directed to the corresponding author/s.
